# Comparative transcriptome profiling reveals distinct regulatory responses of secondary defensive metabolism in *Datura* species (Solanaceae) under plant development and herbivory‐mediated stress

**DOI:** 10.1002/ece3.11496

**Published:** 2024-07-09

**Authors:** Eunice Kariñho Betancourt, Nancy Calderón Cortés, Rosalinda Tapia López, Ivan De‐la‐Cruz, Juan Núñez Farfán, Ken Oyama

**Affiliations:** ^1^ Escuela Nacional de Estudios Superiores (ENES) Unidad Morelia, UNAM Morelia Mexico; ^2^ Laboratorio de Genética Ecológica y Evolución Instituto de Ecología, UNAM Ciudad de México Mexico; ^3^ Laboratorio de Evolución Molecular y Experimental Instituto de Ecología, UNAM Ciudad de México Mexico; ^4^ Department of Plant Protection Biology Swedish University of Agricultural Sciences Alnarp Sweden

**Keywords:** alkaloids, comparative transcriptomics, differential gene expression, herbivory, jasmonate, plant development

## Abstract

Differential expression of genes is key to mediating developmental and stress‐related plant responses. Here, we addressed the regulation of plant metabolic responses to biotic stress and the developmental variation of defense‐related genes in four species of the genus *Datura* with variable patterns of metabolite accumulation and development. We combine transcriptome profiling with phylogenomic techniques to analyze gene expression and coexpression in plants subjected to damage by a specialist folivore insect. We found (1) common overall gene expression in species of similar chemical profiles, (2) species‐specific responses of proteins involved in specialized metabolism, characterized by constant levels of gene expression coupled with transcriptional rearrangement, and (3) induction of transcriptional rearrangement of major terpene and tropane alkaloid genes upon herbivory. Our results indicate differential modulation of terpene and tropane metabolism linked to jasmonate signaling and specific transcription factors to regulate developmental variation and stress programs, and suggest plastic adaptive responses to cope with herbivory. The transcriptional profiles of specialized metabolism shown here reveal complex genetic control of plant metabolism and contribute to understanding the molecular basis of adaptations and the physiological variation of significant ecological traits.

## Graphical abstract

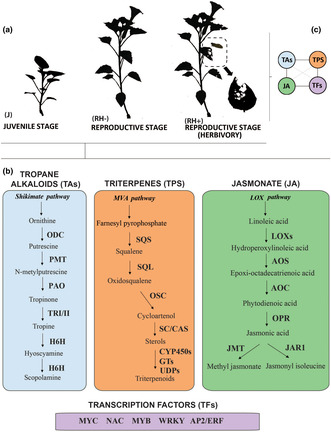

## 1 INTRODUCTION

Plants' secondary metabolism involves highly conserved biosynthetic pathways and complex genetic machinery to produce diverse chemical compounds that play eco‐physiological roles. Secondary metabolites mediate biotic interactions functioning as adaptations to cope with plants' natural enemies, with toxic or deterrent effects on plant consumers (Agrawal et al., 2012; Kariñho‐Betancourt, 2018, 2020). In response to herbivores, a wide array of constitutive and induced *defensive* metabolites is produced by plants, including compounds ubiquitous in green plants that involve large multigene families and enzymes (e.g., terpenes) (Chen et al., 2011; Tohge et al., 2013; Young et al., 1966) or compounds restricted to few plant families that involve a more limited number of enzymes and genes (e.g. alkaloids) (Biastoff et al., 2009; Hanzawa et al., 2002; Wink, 2003).

The metabolic and genetic machinery behind the responses of plants to stress can be triggered by herbivore wounding and/or phytohormone signaling. For instance, it has been shown that phytohormone accumulation, in response to wounding and herbivore‐specific signals, increases the expression of genes coding for enzymatic complexes and transcriptional factors that elicit plant chemical defenses (Agrawal et al., 2002; Kessler et al., 2004; Skibbe et al., 2008); leading to localized or systemic increase in metabolite concentration (Park et al., 2019; Yoshikawa et al., 2018). However, the relationship between wound signaling and gene regulation with the expression of chemical defenses is variable. One elicitor may result in different patterns of transcriptional regulation across different classes of secondary metabolites. For instance, exposure of cell cultures of barrel clover (*Medicago truncatula*) to methyl‐jasmonate results in 50‐fold induction of transcripts encoding terpene enzymes but no induction of phenylpropanoid genes (Suzuki et al., 2005). Although the increase in metabolite accumulation resulting from herbivory is widespread among plants (Boege & Marquis, 2005; del Val & Dirzo, 2003; Jacobo‐Velázquez et al., 2015; Yoshikawa et al., 2018), variable and contrasting developmental trajectories across species, plant organs and chemical traits not associated with biotic stress have been also documented (Brenes‐Arguedas et al., 2006; Goodger et al., 2004), suggesting potential different selective pressures (e.g., resource availability; Endara & Coley, 2011) and regulatory patterns of plant metabolism during the plant's lifetime. These developmental and stress response patterns show how molecular and metabolic changes linked to endogenous hormonal signaling and biochemical cascades shape plant chemical phenotypes (Avanci et al., 2010; Lortzing & Steppuhn, 2016).

One key aspect of understanding the genetic basis of the adaptive response to stress that led to phenotypic variation is to examine interspecific patterns. It has been proposed that the vast phenotypic differences among species are not likely to be explained solely by changes in structural proteins, hence gene regulation is expected to contribute to phenotypic differences between species (King & Wilson, 1975). However, the role of gene regulation in the evolution of phenotypes, including complex traits such as chemical defenses, is poorly understood. Also, even when the role of gene regulation in shaping variation has been associated with species relatedness (Romero et al., 2012; Stern & Orgogozo, 2008), it is currently unclear as to whether common regulatory changes associated with adaptation to stress and development are shared by closely related species or whether there has been divergence. Here, we addressed the transcriptional regulation across congeneric species by comparing the expression of multiple defense proteins along plant development, and in response to wounding by an insect herbivore.

Plant responses to insect herbivores may depend on the degree of ecological specialization of the attacker. For instance, previous studies using *Nicotiana attenuata* have found differential transcriptional and/or chemical responses of plants to herbivores with different degree of specialization (Diezel et al., 2009; Voelckel & Baldwin, 2004). However, other studies using *Arabidopsis thaliana* have failed to find a specific pattern of responses elicited by different herbivore guilds (Mewis et al., 2006; Reymond et al., 2004). During feeding on plant tissue, insects release oral secretions containing a repertoire of molecules that can elicit specific plant defense responses to combat insect attacks (Kallure et al., 2022). While several studies have evaluated plant transcriptional responses to either different feeding guilds or their hormonal elicitors (e.g., jasmonate) (Bidart‐Bouzat & Kliebenstein, 2011), these rarely analyzed the molecular responses across species. Here, we employed the plant genus *Datura* to examine the role of herbivory in the expression of defensive genes by using a specialist insect folivore, the three‐lined potato beetle (*Lema trilineata daturaphila*), as the biotic stressor, across plant species.

Plants of the genus *Datura* are chemically diverse and well‐known for producing tropane alkaloids, which along with triterpenes play a central role as defenses against herbivores. These compounds exhibit large variation during development, which seems to be associated with their adaptive role to cope with diverse herbivore guilds (De‐la‐Cruz et al., 2020, 2021; Kariñho‐Betancourt et al., 2015; Miranda‐Pérez et al., 2016). Across the phylogeny of *Datura*, the specialist herbivore *L. t. daturaphila* has shown to be differentially affected by specific classes of defensive compounds, including major alkaloids scopolamine and hyoscyamine (Kariñho‐Betancourt et al., 2023). Nonetheless, the molecular basis of response to herbivory and their interplay with developmental variation of secondary metabolites has never been evaluated in *Datura*. Hence, species of *Datura* and their specialized herbivore represent good non‐model systems for addressing gene control of defensive secondary metabolism.

We selected four species of *Datura* (*D. stramonium, D. pruinosa, D. inoxia*, and *D. wrightii*) with contrasting developmental changes and chemical phenotypes (i.e., patterns of accumulation of specialized defense‐related compounds) (Table 1) to identify the differential gene expression associated to development and defense. The genus *Datura* has diverged from sister genus *Nicotiana* about 25 Mya, and a recent study on the molecular evolution of the genus suggests that *Datura inoxia* and *D. wrightii* are the most recently derived (~2.3 Mya) species within the *Datura* group (De‐la‐Cruz et al., 2022).

**TABLE 1:** Developmental variation of tropane alkaloids, flowering time, and distribution of four *Datura* spp.

Species	Ontogenetic trajectories^a^ $\left[\delta_{Di}=\left({\bar{S}}_{t_{2}}-{\bar{S}}_{t_{1}}\right)/{\bar{S}}_{t_{1}}\right]$		Average concentration^a^ (μg g^−1^ dry wt)		Distribution (Luna‐Cavazos, 2011)	Approximate time to flowering (weeks)
	Hyoscyamine	Scopolamine	Hyoscyamine at reproductive stage	Scopolamine at reproductive stage		
*D. stramonium*	5.3	2.92	5.3	634.01	Through north and south America, Asia and naturalized in Europe	6
*D. pruinosa*	2.21	0.44	5.88	85.25	Southern Mexico	5–6
*D. inoxia*	53.68	11.16	78.19	2266.82	Central and northern Mexico, India and naturalized in southern Africa	6–7
*D. wrightii*	12.17	12.82	427	1130.75	Northern Mexico, USA and southern Canada	9–12

^a^ From Kariñho‐Betancourt et al. (2015).

**FIGURE 1:**
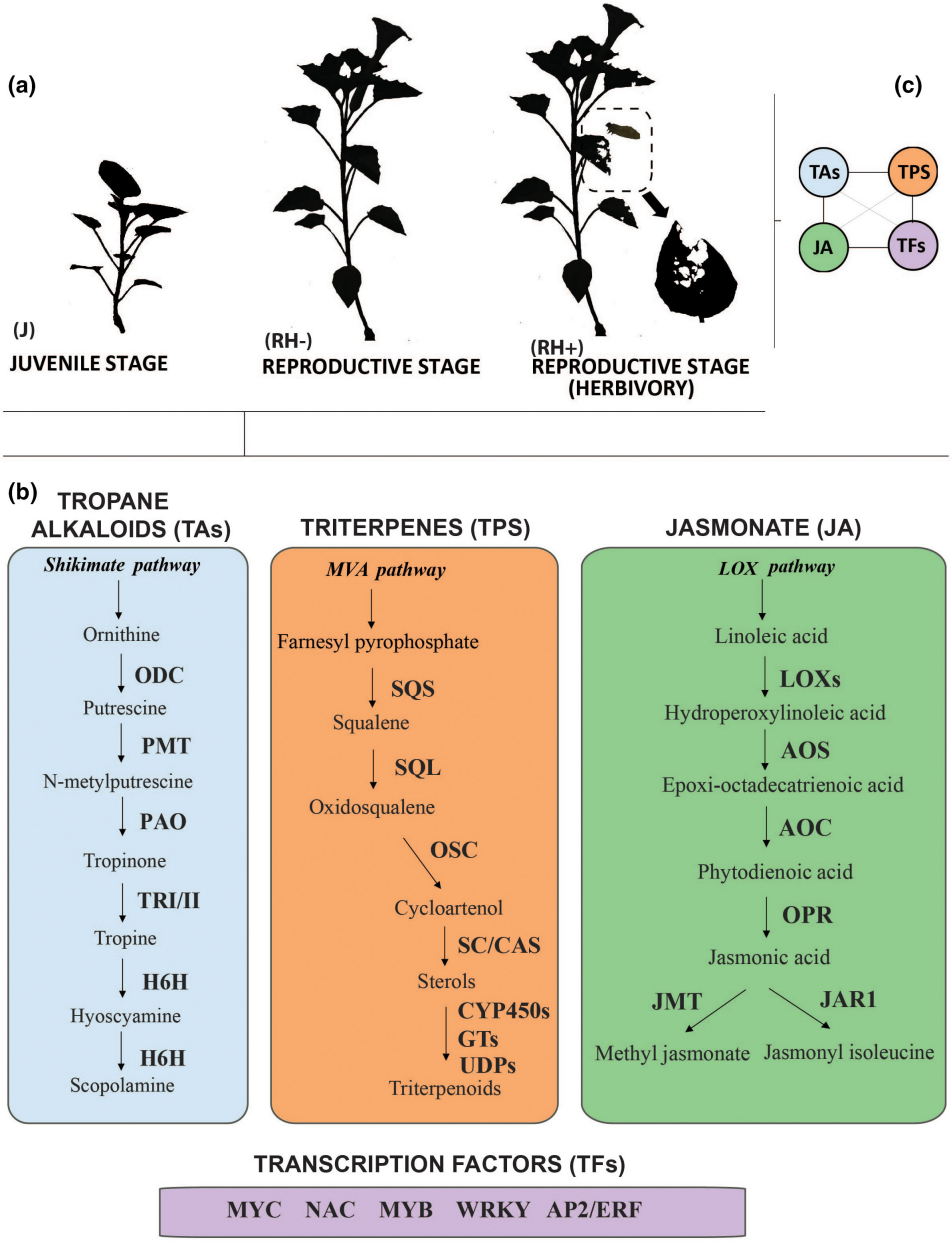
Overview of experimental design and defensive secondary metabolites/proteins examined in *Datura* spp. (a) experimental plants at two developmental stages; juvenile and reproductive. Plants at the reproductive phase were also exposed to the specialist herbivore *Lema trilineata daturaphila*. (b) Main enzymes involved in biosynthesis of TAs, TPS and JA, and related TFs were transcriptionally analyzed across developmental phases. (c) Gene coexpression of four classes of defensive metabolites /proteins was analyzed at the reproductive phase. Enzyme names are as follows: TAs biosynthesis: H6H, Hyoscyamine‐6‐dioxygenase; ODC, Ornithine decarboxylase; PAO, Polyamine oxidase; PMT, Putrescine N‐methyltransferase; TRI/II, Tropinone reductase I/II. TPS biosynthesis: CYP450s, cytochrome P450s; GTs/UDPs, glycosyltransferases; OSC, oxidosqualene cyclase; SC/CAS, cycloartenol synthase; SQL, squalene monooxygenase; SQS, squalene synthase. JA biosynthesis: AOC, allen oxide cyclase; AOS, allene oxide synthase; JAR1, jasmonate amido synthase; JMT, jasmonate O‐mehyltransferase; LOXs, lipoxygenases; OPR, oxo‐phytodienoic acid reductase.

Here, we report the differential gene expression across species of specialized metabolite classes: tropane alkaloids (TAs), terpenes (TPS), jasmonate (JA) and their transcription factors (TFs) at two different developmental plant stages. We also analyzed gene expression and coexpression in plants subjected to damage by the specialist folivore (Figure 1). Specifically, we asked the following questions: Are there common regulatory patterns related to plant chemical defenses among *Datura* species? How do different defense‐related proteins vary among species in response to herbivory and plant development? And which genes control the induced defensive responses?

## 2 MATERIALS AND METHODS

### 2.1 Experimental design and plant material

We selected four *Datura* species, *D. stramonium, D. pruinosa, D. inoxia*, and *D. wrightii*, with different developmental and contrasting patterns of alkaloid accumulation (i.e., variation in concentration at different developmental stages). Although most species germinate during spring or early summer and begin flowering approximately 2 months after germination (Table 1), the growth rate and development may vary along the distribution range. Here, we selected two fast‐growth species, *D. stramonium* and *D. pruinosa*, characterized by low concentrations of tropane‐based metabolites, and a pair of slow‐growth species, *D. inoxia* and *D. wrightii*, that accumulate a larger amount of tropane metabolites (see Table 1).

Replicate plants (nine maternal families) of each of the four species were grown from seeds in a glasshouse. All plants were planted in 150‐mL pots, in sterilized soil and watered ad libitum. Plants were grown under a 16:8 L:D cycle at 25°C:20°C (L:D). Fully expanded leaves from each plant were harvested at juvenile and reproductive stages. Empirical evidence suggests that variation of chemical compounds, especially alkaloids, within and among populations, along development, and across *Datura* species is related to changes in herbivore composition and their abundance, which increases at flowering (Castillo et al., 2013, de‐la‐Cruz et al., 2020, Kariñho‐Betancourt et al., 2015). Hence, to capture ecological dynamics, when plants reached 15 cm or had at least two branches (~ 1 month after germination) at the juvenile stage (J), three juvenile plants per species were defoliated, whereas the other six plants remained undamaged until flowering. When the first flower emerged, at the reproductive stage (~ 2 months after germination), three of the nondamaged plants were assigned to the herbivore treatment (RH^+^) and the other three remained undamaged (RH^−^) (Figure 1a). Once the first flower fully expanded, plants were exposed to larvae of the specialist folivore of *Datura*, the three‐lined potato beetle *Lema trilineata daturaphila* (Coleoptera: Chrysomelidae). On each leaf (10 leaves per plant), two larvae of second to fourth instar were randomly placed at the adaxial side of fully expanded leaves. After 48 h, larvae were removed and all damaged leaves were collected. At the same time, 10 leaves of each undamaged plant were harvested. Leaves were flash frozen and stored at −80°C.

### 2.2 Sequencing, transcriptome assembly, and functional annotation

**FIGURE 2:**
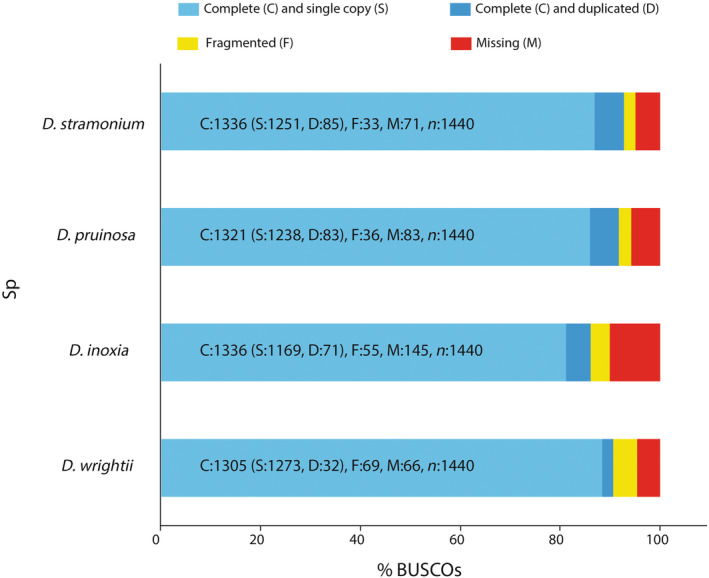
BUSCO scores of four *Datura* transcriptomes. The plot shows quantitative measures for the assessment of relative completeness based on conserved gene sets from nearly‐universal single‐copy orthologs selected from the “embryophyta_odb9” database.

Total RNA of each individual plant was extracted from the leaves of four *Datura* species using the TRIzol extraction method (Rio et al., 2010). (dx.doi.org/10.17504/protocols.io.bx4zpqx6). RNA quality and quantity were determined using a Nanodrop 2000 instrument (Thermo Scientific) and Bioanalyzer Chip RNA 7500 series II (Agilent). Thirty‐six libraries were prepared using total RNA (fragment size of 500 bp). Samples were sequenced using an Illumina NextSeq 500 under a paired‐end 2 × 75 mode. We generated 333.3 M (millions) paired‐end raw sequences, 93.4 M sequences on average by species and 244.4 M total counts. The raw read data from Illumina sequencing for each species were deposited in the NCBI under BioProject PRJNA669339. The quality and contamination levels of RNA‐seq reads were verified using FastQC (Andrews et al., 2010), and Trimmomatic (Bolger et al., 2014) was used to remove sequences of ≤20 Phred quality score. Leaf transcriptomes of each species were de novo assembled using Trinity (trinitymasq −2.0.6) (Grabherr et al., 2013), combining paired‐end reads from nine samples (J, RH^−^ and RH^+^ plants). For each species, we produced de novo assemblies that yielded a total of 413,241 transcripts. We evaluated the transcriptome assemblies using standard assembly statistics (total genes and transcripts, percent of GC, Nx length statistics and median and average contig) with the script “TrinityStats.pl” of Trinity v2.11.0 (Grabherr et al., 2011; Haas et al., 2013). The longest isoform of each component was extracted for downstream analyses. We obtained 33,851 transcripts for *D. stramonium*, 30,191 for *D. pruinosa*, 26,122 for *D. inoxia* and 30,385 for *D. wrightii* (Table 2) The relative completeness of transcriptomes was evaluated by using Benchmarking Universal Single‐Copy Orthologs (BUSCO). We performed BUSCO v.3.0.2 analysis with the transcriptome mode option and the lineage set to embryophyta_odb9 (Simão et al., 2015). The *Datura* transcriptomes ranged between 86% and 92% relative completeness (Figure 2). In comparative studies, the transcriptome completeness is fundamental for the detection of orthologous genes, and the uneven distribution of (transcripts) gene copies is usually indicative of the differences in gene family sizes and does not restrict comparisons among species. In our study, the BUSCOs ranging indicates high‐quality assembly and annotation, and suggests good relative completeness. Each assembly was functionally annotated using the Trinotate v3.2.1 pipeline (Bryant et al., 2017). We used Transdecoder v5.5.0 (https://github.com/TransDecoder/TransDecoder) to find putative protein‐coding sequences (CDS) in each transcriptome. Each set of CDS and translated amino acid sequences was then annotated using blastp v2.5.0 with a maximum e‐value of 1e‐5 (Altschul et al., 1997) and HMMER v3.3's hmmscan with default settings against the Swiss‐Prot (Boeckmann et al., 2003) and Pfam (Bateman et al., 2002) databases.

**TABLE 2:** Trascriptomic statistics of four *Datura* species.

Species	# of total Illumina sequences	# of total assembled transcripts (trinity)	# of total genes (trinity)	% of GC	N50 (longest isoform per gene)	# of transcripts/genes after polishing (longest isoform)
DS	86,626,733	113,343	76,635	40.18	1511	33,851
DI	104,940,331	81,748	51,776	40.1	1683	26,122
DW	89,135,921	111,196	68,353	40.3	1546	30,385
DP	94,261,673	103,954	66,186	40.28	1589	30,191

Abbreviations: DI, *Datura inoxia*; DP, *Datura pruinosa*; DS, *Datura stramonium*; DW, *Datura wrightii*.

### 2.3 Differential gene expression

Read counts per component were estimated using the RSEM software package, and differential gene expression was assessed using edgeR 3.24.3 (Chen et al., 2014; Robinson et al., 2010). Here, we present the results of the significant differential expression [false discovery rate (FDR) <0.05, *p* ≤ .01, log2‐fold change (FC) ≥ 1] from two contrasts between treatments per species; Contrast 1 (C1; RH^−^ vs. J) and Contrast 2 (C2; RH^−^ vs. RH^+^), showing either changes due to plant development (C1) or herbivory (C2). To identify defense‐related genes/transcripts, we used custom scripts in R for data mining R (Wickham, 2016) and searched for proteins of biosynthetic pathways involved in the expression of tropane alkaloids, terpenes, jasmonate, and transcription factors within species (Figure 1b,). We constructed heat maps using the full set of differentially expressed metabolic genes using the gplot library in R (R Development Core Team 2014; Wickham, 2016), and we use the average of log2TPM (Lin & Pang, 2019) to display the expression data for each of the three conditions (juvenile, reproductive without damage, reproductive with herbivory).

### 2.4 Orthologous identification and clustering analysis

We employed the set of differentially expressed metabolic genes across four *Datura* species for identification of orthologous genes (orthologs). Orthologs are of particular interest because they can be expected to have maintained at least part of their (ancestral) biological function (Lechner et al., 2011) and allow the identification of common molecular mechanisms across taxa. Protein‐coding genes and CDS from two sister species, *Nicotiana tabacum* and *Solanum lycopersicum*, were sourced from the Sol Genomics Network2. We used the combined dataset of *Datura*, and *Nicotiana* and *Solanum* proteomes to infer orthogroups in Proteinortho v 6.5 program (Lechner et al., 2011). In addition, to identify species linkage, we performed a hierarchical clustering analysis (Nielsen, 2016), employing orthology scores and overall number of differentially expressed genes, and the count of each metabolic class by species. We employed the Euclidean distance between samples and the complete linkage method (“ward”) for clustering (Nielsen, 2016).

### 2.5 Gene coexpression network analysis

We parsed the expression profile (significantly differentially expressed genes) and selected (212) genes differentially expressed in at least one treatment group across three *Datura* species (*D. stramonium, D. pruinosa*, *and D. wrightii*). To identify genes controlling the response to herbivory we used the parsed database of normalized expression profiles of each species to carry out a pairwise Pearson correlation analysis. For networks construction, only significant correlations (*p* < .05) were retained using the False Discovery Rate method (Benjamini & Hochberg, 1995). Coexpression networks help to identify relationships and discover key regulatory elements. The program Igraph v1.2.6 (Csardi & Nepusz, 2006) was used to construct the networks by species. Network visualizations were performed using Cytoscape v3.8.2 (Shannon et al., 2003). The application clusterMaker (community clustering with default options) was used in Cytoscape to carry out a network topology analysis (i.e., the structure that determines how genes are connected) (Contreras‐López et al., 2018; Csardi & Nepusz, 2006).

## 3 RESULTS

### 3.1 Overall differential gene expression

**FIGURE 3:**
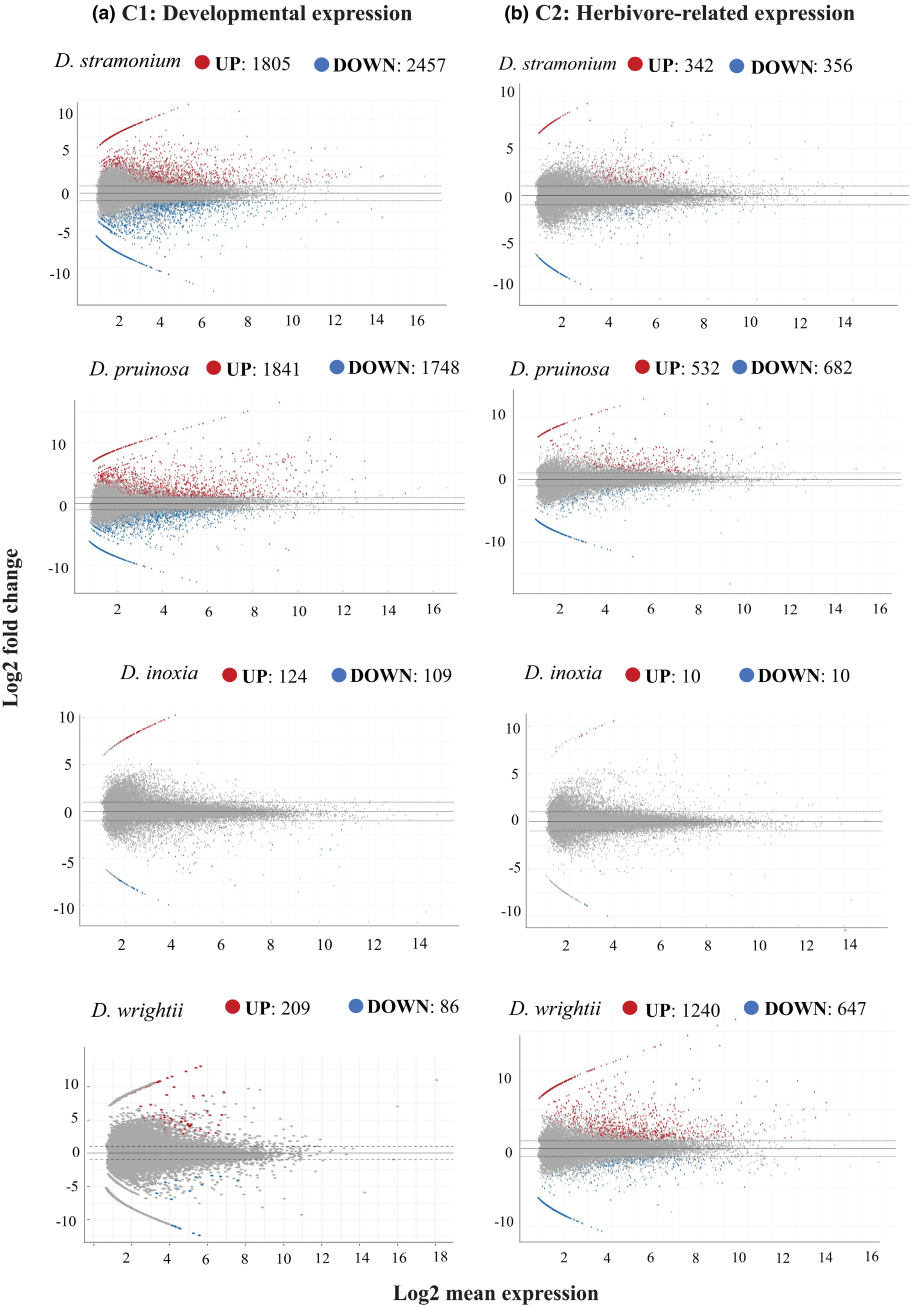
MA plots of leaf transcriptomes of four *Datura* species at different developmental phases and exposed to different biotic environments. Panels at left side (a) depict the differential gene expression (FC ≥ 1, FDR < 0.05 y *p* < .01) from the contrast C1 (undamaged reproductive plants vs. plants at juvenile stage). Panels at the right side (b) depict the expression from the contrast C2 (undamaged plant at the reproductive phase vs. reproductive plants expose to the herbivore *Lema trilineata daturaphila*).

We found 12,234 differentially expressed genes across the four *Datura* species, during development (C1) and in response to herbivory (C2). Overall, differential expression was larger during development, comprising 69% (8415) of the differentially expressed genes. However, the pattern of gene regulation differed between development and herbivore‐related contrasts. At C1, downregulated genes represented 53% of the differential expression, whereas at C2 upregulated genes represented 56% of genes. Although most species, including *D. stramonium, D. pruinosa*, and *D. inoxia*, showed a larger fraction of differentially expressed genes during development, *D. wrightii* showed the opposite pattern (Table 3). At C2, *D. wrightii* registered 1240 upregulated genes, ca. 9‐fold difference of upregulated genes at C1 (Figure 3a). In contrast, *D. stramonium* and *D. pruinosa* registered a higher differential expression at C1 with 3‐5‐fold upregulated genes compared to C2. Although *D. inoxia* showed the least number of differentially expressed genes, representing the 2% of total expression (Table 3) during development, *D. inoxia* and *D. wrightii* expressed nearly the same number of genes (Figure 3b).

**TABLE 3:** Summary of significant differential gene expression [false discovery rate (FDR) <0.01, *p* ≤ .05, log2‐fold change (FC) ≥ 1] of four *Datura* transcriptomes.

	C1 (developmental expression)	C2 (herbivore‐related expression)	% from total genes
*D. stramonium*	4262	698	41
*D. pruinosa*	3589	1214	39
*D. inoxia*	233	20	2
*D. wrightii*	295	1887	18

*Note*: Gene expression of two contras C1 (reproductive vs. juvenile plants) and C2 (Reproductive undamaged plants vs. Reproductive plants exposed to the herbivore *Lema trilineata daturaphila*) is shown.

### 3.2 Defense‐related genes: Expression and regulation

**FIGURE 4:**
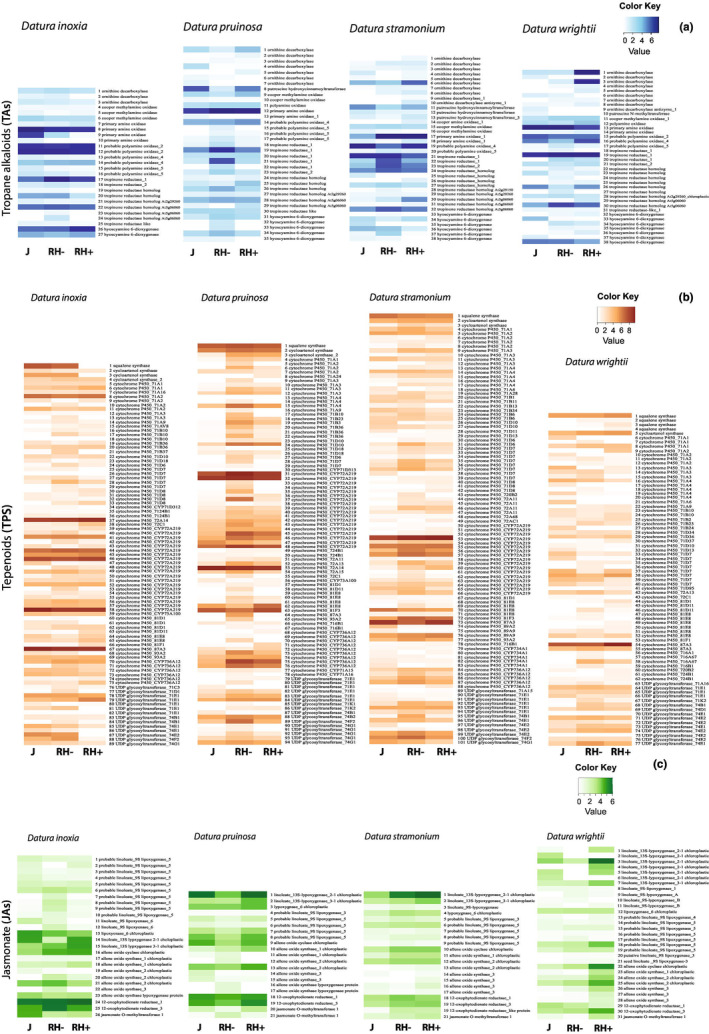
Heatmaps of defense related genes (*N* = 598) of four *Datura* species. Shown are the mean of TPM normalized log2 transformed counts of genes related to the synthesis of (a) tropane alkaloids, (b) terpenes and (c) jasmonate. Plants were examined at different developmental stages and exposed to the specialized herbivore *Lema trilineata daturaphila*. Columns represent each experimental group. J; juvenile, RH−; reproductive undamaged and RH+; reproductive exposed to herbivory.

We identified 5135 *TA*s, *TPS*, *JA*, and *TF*s defense‐related genes in the four *Datura* species. Terpene genes had the largest annotation (60%) followed by transcription factors (20%), jasmonate genes (13%), and tropane alkaloids (7%). Annotation of defensive genes was similar among species, from 1120 genes (*D. inoxia*) to 1417 genes (*D. wrightii*). The expression of the different classes of metabolic genes was highly variable between species and juvenile (J), reproductive with (RH+) and without herbivory (RH‐) plant stages. Each metabolic class showed either contrasting expression at juvenile (J) and reproductive (RH) stages or a steady expression across all treatments. For instance, in all species, specific *TA*s and *TPS*, such as amine oxidases and cytochrome of the subunit 72, were highly expressed across all treatments (Figure 4b). Likewise, genes of the alkaloid metabolism, including amino oxidases (PAO) and tropinone reductase (TR‐I), showed a high expression along treatments in all species. By contrast, the highest expression of genes coding for key enzyme hyoscyamine 6‐dioxygenase (H6H) was detected in *D. inoxia* upon herbivory (Figure 4a). Likewise, key jasmonate genes for plant defense such as the jasmonate O‐methyl transferase (JMT) and lipoxygenase (LOX) genes were highly expressed in *D. wrightii* (Figure 4c).

**FIGURE 5:**
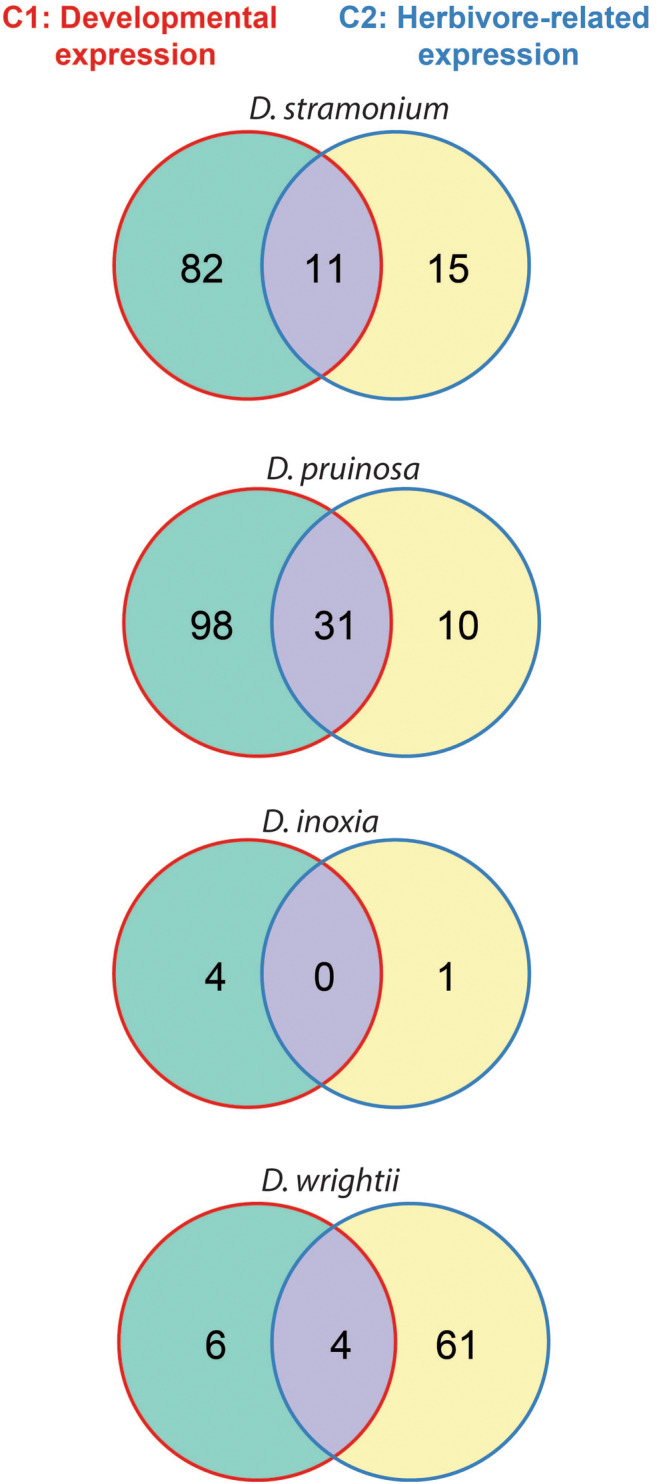
Venn diagrams of differentially expressed genes (log2 fold change ≥1, *p*‐value <.01, FDR < 0.05) depicting developmental‐ and defense‐related expression in four *Datura* species. Plants were examined at different developmental stages and exposed to the specialized herbivore *Lema trilineata daturaphila*. Gene expression was contrasted along ontogeny (C1: undamaged reproductive plants vs. plants at juvenile stage) and in response to herbivory (C2: undamaged reproductive plants vs. reproductive plants exposed to herbivores). Shown are counts of genes (*N* = 323) associated with the *defensive* metabolism of tropane alkaloids, terpenoids, jasmonate and specific transcription factors.

While expression (number of annotated genes) of defensive proteins was comparable among species, gene regulation differed. We identified 323 differentially expressed genes associated with different classes of metabolites and transcription factors. The largest differential expression of defense‐related genes with 43% of proteins was recorded in *Datura pruinosa* (139 genes), followed by *D. stramonium* (108 genes), *D. wrightii* (71 genes), and *D. inoxia* (5 genes). About 6%–20% differentially expressed genes were shared by both the development‐ and herbivore‐related contrasts within species. However, *D. inoxia* did not share any gene between treatments (Figure 5). The pattern of regulation varied among species and between contrasts. The developmental contrast captured most of the differential expression of defensive genes. *Datura stramonium*, *D. pruinosa*, and *D. inoxia* comprised a larger differential expression during development (C1) (development contrast), with 92, 129 and 4 genes, respectively. By contrast, *D. wrightii* showed a larger response to herbivory registering 61 genes at C2 that represents 4‐fold genes than *D. pruinosa*, which had the highest number of differentially expressed genes (Figure 5).

#### 3.2.1 Gene regulation across species

**FIGURE 6:**
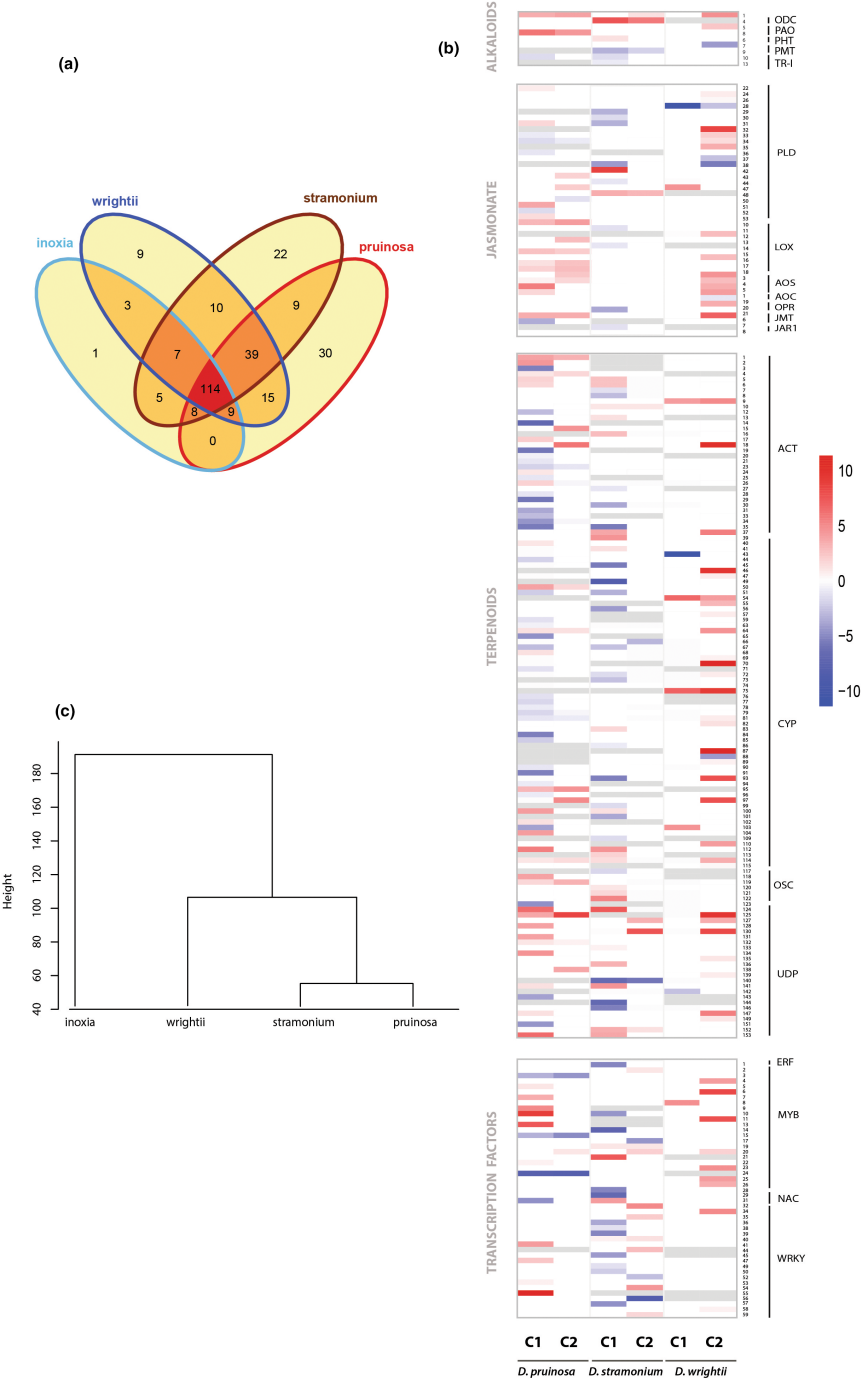
Differential gene expression (log2 fold change ≥1, *p*‐value <.01, FDR < 0.05) of defense‐related genes involved in the alkaloid, jasmonate and terpenoid metabolism, and specific transcription factors, in the genus *Datura*. Plants were examined at different developmental stages and exposed to the specialized herbivore *Lema trilineata daturaphila*. Gene expression was contrasted along ontogeny (C1: undamaged reproductive plants vs. plants at juvenile stage) and in response to herbivores (C2: undamaged plant at the reproductive stage vs. reproductive plants expose to the herbivory). (a) Venn diagram of species‐specific and common (orthologous) genes (*N* = 281) among four *Datura* species that were differentially expressed in at least one contrast. (b) Heat map of 219 differentially expressed genes across three *Datura* species. Each row represents an orthologous gene (shared by at least two species) related to the synthesis of defensive secondary metabolites and transcription factors. Shown are differentially expressed genes from the contrasts C1 and C2. Gray bars indicate absence (no detection based on the orthology analysis) of a gene. (c) Hierarchical clustering of four *Datura* species. The dendrogram depicts the result of hierarchical clustering based on the species‐specific and overall number of differentially expressed genes, and the count of each metabolic class. Euclidean distance between samples and the complete linkage method (“ward”) were used for clustering. See Table S1 for the full gene names.

We analyzed the orthologous genes associated with the defensive metabolism of four *Datura* species to identify common regulatory responses. We found 281 orthologous genes across all species that were differentially expressed in at least one species. About 40% of these genes occurred in all species and 22% were species‐specific (Figure 6a). *Datura pruinosa* and *D. stramonium* had the highest number of species‐specific genes and shared most of the defense‐related orthologous. The metabolism of alkaloids accounted for nine orthologous genes associated with five different enzymes, including upstream (early signaling in a metabolic pathway) and downstream (late signaling implicate in synthesis of final products) complexes (Figure 6b). The highest number of orthologous genes was detected for the terpenoid metabolism with 157 genes. The jasmonate metabolism registered 52 genes. Although the pattern of regulation of these metabolic genes was highly variable across species and contrast, overall, upstream genes were downregulated during development. For instance, phospholipases (PLD) genes of the jasmonate metabolism showed the largest downregulation (*D. wrightii*) with −10.8 logFC (Table S1). Likewise, most acyltransferase (ACT) genes were downregulated in *D. pruinosa*, and they did not express differentially in the rest of the species. By contrast, genes of downstream enzymes were upregulated in most species showing the highest levels of change in response to herbivory. For example, the jasmonate O‐methyltransferase (JMT) gene, key for plant defense signaling, was upregulated in both *D. pruinosa* and *D. wrightii*, showing the largest change in *D. wrightii* (7.37 logFC) in the herbivore contrast. Terpenoid genes, including the CYP and downstream UDP enzymatic complexes, were upregulated in both contrasts but showed the largest changes at the herbivore‐related contrast. For instance, dolichyl‐diphosphooligosaccharide‐protein_glycosyltransferase and glycosyltransferase_family_protein_64_C3 associated with UDP enzymes showed a range of 6.6 to 8.6 logFC across three *Datura* species (*D. stramonium, D. pruinosa*, *and D. wrightii*). Additionally, we found 59 orthologous genes related to transcription factors, NAC and WRKY, that were downregulated during development in *D. stramonium* and not differentially expressed in the rest of the species, contrasting with MYB genes that were mostly upregulated in *D. pruinosa* and *D. wrightii* (Figure 6b). *Datura wrightii* not only showed some of the most substantial upregulatory changes (about 90% of genes were upregulated), but most of these changes occurred solely in response to herbivores.

Based on the rates of differential expression of each species, the clustering analysis showed similarities in regulation of metabolic defensive genes across species. Two major groups were observed across treatments: the fast‐growth species *D. stramonium* and *D. pruinosa* clearly separated from the slow‐growth species *D. inoxia* and *D. wrightii* (Figure 6c).

### 3.3 Gene coexpression of defensive metabolites in response to herbivory

**FIGURE 7:**
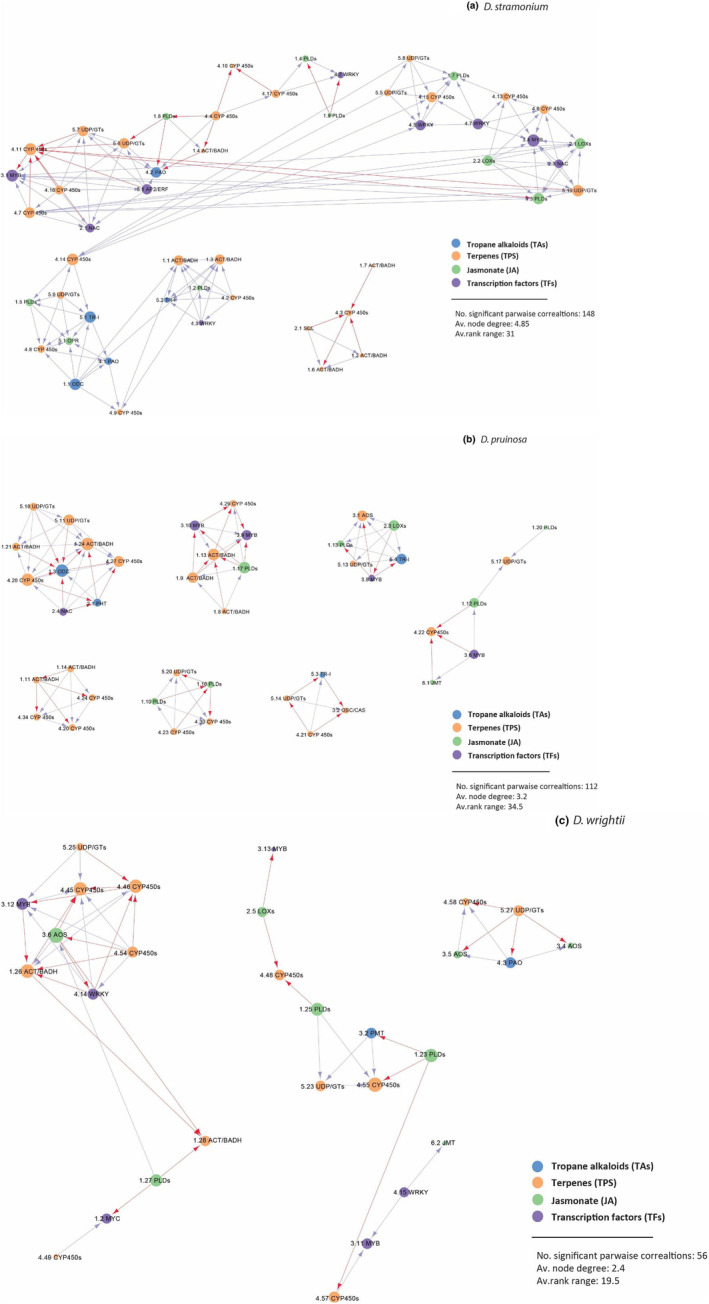
Gene coexpression networks of differentially expressed genes of three *Datura* species; *D. stramonium* (a), *D. pruinosa* (b) and *D. wrightii* (c). The node represents one gene of chemical classes TAs, TPS, JA or TFs, and the edge between nodes represents the subordinate relationship between nodes with arrow from the lower ranking gene pointing towards to the higher one. Red arrows indicate negative correlations and blue arrows indicate positive correlations. Larger size nodes are highly connected “hub” genes (>average node range). For the full names of genes, see Table S2.

To examine gene control in response to herbivory, we employed a dataset of 212 differentially expressed metabolic genes in at least one treatment group across three *Datura* species (*D. stramonium, D. pruinosa*, and *D. wrightii*) in plants exposed to the larvae of the three‐lined potato beetle (*L. t. daturaphila*). The analysis of coexpression produced variable networks of either connected genes and unconnected modules. In total, 24 modules containing more than three genes (nodes) were generated. Averaging across the three coexpression datasets, the number of genes assigned to modules ranged from 5 to 21 (Table S2), and the average of significant pairwise correlations was 105.3. The analysis estimated 79 highly connected hub genes (above the average node degree per network), which control the network. Top common hub genes across *Datura* species included major terpene gene families of *CYP450*s and *ACT/BAHD*, as well as *MYB* transcription factor genes. We found large variation in shape and size of networks among species. *Datura stramonium* showed the largest network (148 connected genes) comprised the greatest number of hub genes (39), including common *TPS* genes, *MYB and WRYK*, and *TA*s genes such as *ODC, PAO*s, and *TR‐I*. The top *TPS* were negatively correlated with *TF*s, but most *TA*s were positively correlated with *TPS* and most *TF*s genes (Figure 7a). *Datura pruinosa* comprised 122 significant correlations and showed the highest number of modules, which were all unconnected and controlled by 17 hub genes. In addition to *TPS*, major hub genes of *D. pruinosa* included *TA* genes such as *ODC* and *TR‐I* and several *JA* genes (*AOS, LOX, PLD*s). *TA* hub genes were negatively correlated with major *TPS* and *JA* hub genes (Figure 7b). *Datura wrightii* comprised the smallest network (56 significant correlations) and the least connected genes arranged in six modules. This species had 19 hub genes, including common *TPS*, and *AOS* and *PLD* genes of jasmonate synthesis that were negatively correlated with each other. *TA*s included *PMT* genes that coregulated *TPS* and *JA* genes (Figure 7c).

## 4 DISCUSSION

This study examined and compared the expression and regulation of metabolic genes in response to herbivory and along plant development in four *Datura* species. Our results revealed common patterns of gene differential expression in species of similar chemical profiles and growth rates. Common orthologues to four species involved one‐third of the differentially expressed metabolic genes of *TA*s, *TPS*, *JA*, and *TF*s, whose major regulatory changes were given by the terpenoid and jasmonate metabolisms. Species‐specific responses were characterized by constant levels of gene expression coupled with transcriptional rearrangement in both developmental‐ and herbivore‐related pathways. We also found that the regulation of major *TPS* and *TF*s associated with certain *JA* genes controls the metabolic induced response to herbivory in *Datura* species, but key *TA*s coregulate species‐specific responses.

### 4.1 Regulatory patterns across species: Developmental‐ and defense‐related patterns

We analyzed gene regulation among two fast‐growth (flowering within 6 weeks) species, *D. stramonium* and *D. pruinosa*, and two slow‐growth (flowering between seven and 12 weeks) species, *D. inoxia* and *D. wrightii*. We found contrasting overall differential gene expression between fast‐growth and slow‐growth species. Whereas the expressions of *D. stramonium* and *D. pruinosa* were greater during development involving ca. 2 to 8‐fold genes than in response to herbivory, differential expression of slow‐growth species that can be facultatively biannual, such as *D. wrightii*, showed the opposite pattern with ca. Ninety percent genes implicated in the response to herbivory. These results suggest that molecular changes, including gene regulation and transcriptional rearrangement, are differently driven during plant development and by biotic stress and might be also influenced by plants' growth rate and lifespan. Developmental dynamics in short‐lived annual plants implicate large transcriptional changes that enable fast homeostasis restoration and promote cell survival (Baena‐González & Sheen, 2008). For instance, during development of annual grasses, such as maize, up to 64% of differentially expressed genes are involved in the bundle sheath (Li et al., 2010), including expression of large gene families mostly implicated in cell proliferation. By contrast, in the annual species *Arabidopsis thaliana*, gene expression in response to biotic stress (wounding) represented about 1% of expressed genes (Klepikova et al., 2016). Yet, besides lifespan, transcriptional responses to environmental stress involve a variable number of genes. For instance, while the annual tomato species, *Solanum lycopersicum* exhibited 169 differentially expressed genes in response to infestation of whitefly *Bemisia tabaci* (Estrada‐Hernández et al., 2009), the long‐lived tree *Eucalyptus melliodora* showed less than 30 genes implicated in chemical defense against herbivores (Padovan et al., 2013). These changes in gene control and altered gene expression (up and downregulation) in response to herbivory are particularly evident when focusing on genes associated with ecological relevant strategies (e.g., life‐history traits).

#### 4.1.1 Defensive metabolism: Common regulatory patterns

Orthologous relationships among defense‐related metabolic proteins of tropane alkaloids (TAs), terpenes (TPS), jasmonate (JA), and transcriptional factors (TFs) were found across four *Datura* species. About one‐third of the differentially expressed metabolic genes were shared by the four *Datura* species, including large gene families of terpenes and phytohormones that are highly conserved and diversified in green plants (Butler et al., 2018; Nieuwenhuizen et al., 2013), as well as lineage‐specific tropane genes. In *Datura*, orthologous *TPS* and *JA* genes have been implicated in plant development and evolution of plant defense (De‐la‐Cruz et al., 2022). In diverse Solanaceous species, transcriptional reprogramming of *TPS* orthologous and specific TFs are responsible for widespread resistance responses against herbivory (Smith et al., 2014). Hence, the expression of defense‐related orthologous genes documented in our study suggests common mechanisms to actively modulate growth and reprogram transcriptional regulatory responses upon herbivory.

Based on gene orthology, we found diverse clustering among defensive proteins within the genus. Species of similar growth rates and chemical profiles showed comparable patterns of regulation sharing the most differentially expressed genes. Terpene and jasmonate metabolism were more extensively expressed. Diverse proteins of the large gene family of Cytochromes P450 were differentially expressed across four *Datura* species, mostly upregulated in response to herbivory in *D. wrightii*. Cytochrome genes, especially the subunits *CYP71* and *CYP72*, mediate the expression of terpenes, which are implicated in several ecological and physiological functions. It has been shown that overexpression of *CYP*s regulates sterol metabolism driving defense responses to wounding (Awasthi et al., 2015; Nahar et al., 2017). Overlapping patterns of regulation among species in our study also involved the extensive differential expression of *JA* genes including families of phospholipases (PLDs) and lipoxygenases (LOXs) that play substantial roles during all stages of plant life such as seed germination, growth, development and response to environmental stress such as salinity (Shaban et al., 2018). These jasmonate genes were highly abundant suggesting broad physiological and ecological functions. Nonetheless, our results support a main defensive role of jasmonate metabolites since the highest levels of expression of specific jasmonate genes related to major enzymes were observed after herbivory. Elevated expression levels of *JA* and upregulation of *LOX* have been detected in defensive responses (Hasegawa et al., 2011; Mazur et al., 2018; Wang et al., 2008). It has been argued that wounding causes the release of linoleic acid (the presumed precursor of JA) and the induction of molecular mechanisms that deter insect feeding (Farmer et al., 1992; Turner et al., 2002) and enhance healing. Wounding also induces transcription factors that regulate jasmonate metabolism, including MYB and WRKY59 (Zhou & Memelink, 2016). Our analysis confirms the central role of hormone signaling and their interplay with transcription factors in response to leaf damage by herbivory.

#### 4.1.2 Defense‐related metabolism: Species‐specific responses

Differential expression of metabolic genes involved in the herbivory response included less than one‐third of genes expressed during development in the species of common chemical profiles (i.e., low concentration of alkaloids), *D. stramonium* and *D. pruinosa*. In addition, in these species, a substantial fraction of genes (10%–20%) regulate both the developmental and stress responses. Previous studies have shown that similar genes respond to environmental cues and some may also regulate development (Cooper et al., 2003). Genes implicated in phytohormone signaling and transcription factors control a wide range of physiological processes and mediate plant stress responses due to their ability to elaborate complex networks and crosstalk (Verma et al., 2016). For instance, it has been shown that overexpression of WRKY regulates expression of *JA* leading to induction of plant defenses (Li et al., 2004). In our study, jasmonate genes, including several lipoxygenases (LOX), jasmonate O‐methyltransferases (JMT), and WRKY and MYB transcription factors, were differentially expressed in developmental and stress pathways (i.e., increasing expression at juvenile stage and in response to herbivores), suggesting that synergistic interactions of plant hormones with transcription factors play a relevant role in helping plants to grow and face biotic stress.

*Datura inoxia* and *D. wrightii*, characterized by accumulating large amounts of major tropane metabolites (including hyoscyamine and scopolamine), showed an opposite pattern of gene expression. Regulation in development and in response to herbivory were practically independent, since only *D. wrightii* shared a small fraction of genes (about 5%) between the two pathways. Also, in *D. wrightii*, differential expression of the herbivory‐related response was 6‐fold higher than that of development, doubling the number of upregulated genes. The transcriptional rearrangement of *D. wrightii* mainly occurred in the terpene and jasmonate metabolism, with key genes showing the highest expression of the study, including the cytochrome P450 72A219, 12‐oxophytodienoate reductase, and jasmonate O‐methyltransferase. The upregulation of *JMT* is strongly implicated in the induced synthesis of several defensive metabolites, including diverse terpenes (Ling et al., 2020; Reim et al., 2020). Our results indicate that expression of specific terpenoid genes linked with simultaneous jasmonate signaling can be locally induced by herbivore feeding, supporting the empirical evidence in natural populations of *Datura* on the defensive role of terpenoids against specialist herbivores, including *Lema trilineata daturaphila* (De‐la‐Cruz et al., 2020).

By contrast, the expression of several tropane genes was mostly uniform throughout development and under herbivory in most *Datura* species. Previous studies in the sister genus *Nicotiana* have shown that alkaloid induction is linked to jasmonate signaling and to upregulation of upstream tropane genes after leaf wounding (Guo et al., 2021; Shoji et al., 2000). Yet, the expression of genes coding for key tropane enzymes such as putrescine N‐methyltransferase (PMT), which triggers the synthesis of tropane alkaloids, may remain unchanged after leaf damage (Sinclair et al., 2004). Accordingly, our data showed that key tropane genes such as *PMT* or *TR‐I* did not experience significant transcriptional changes upon herbivory, even in species of high alkaloid concentration such as *D. wrightii* and *D. inoxia*. However, high concentration of major alkaloids hyoscyamine and scopolamine previously documented in these species may result from continuous levels of high gene expression instead of induction. In a recent study that examined the performance of specialist and generalist herbivores in the genus *Datura*, it was documented that *D. inoxia* is one of the few species of the genus in which *L. t. daturaphila* performs poorly (Kariñho‐Betancourt et al., 2023), suggesting a strong negative effect of defensive alkaloids. Notably, even when almost no differential expression was detected in *D. inoxia*, this species showed the highest expression of *TR‐I* and *H6H*, suggesting that the highest relative concentration of hyoscyamine documented for the genus (Kariñho‐Betancourt et al., 2015) is not associated to transcriptional changes. Tropinone reductase I (TR‐I) and hyoscyamine‐6‐dioxygenase (H6H) are key enzymes catalyzing the downstream reactions for the synthesis of ending products of alkaloid biosynthesis, hyoscyamine, and scopolamine (Dräger, 2006), both implicated in plant defense against a diverse array of herbivores (Castillo et al., 2013; De‐la‐Cruz et al., 2021; Shonle & Bergelson, 2000). These results suggest that responses of plants to stress rely not only on gene/metabolite induction but also on maintaining constitutive levels of defenses.

### 4.2 Gene control of plant defensive responses to herbivory

We analyzed gene coexpression in three species of *Datura* (*D. stramonium*, *D. pruinosa* and *D. wrightii*) exposed to the three‐lined potato beetle that showed significant levels of differential expression on metabolic defensive genes. *Datura inoxia* was not included in the analysis because it showed differential expression in only five genes. The analysis revealed that herbivore stress can lead to species‐specific changes in structure (size and connectedness) of gene coexpression networks. For instance, the networks of *D. stramonium* and *D. pruinosa* were similar in size, but differed in shape and connectedness, revealing different relationships of modules (gene clusters) for each species. We observed the smallest number of nodes in *D. wrightii*, whose network was composed of fragmented modules that were mostly expanded. Previous studies have shown that stress leads to increased clustering (increased susceptibility to fragmentation and larger diameters) (Lehtinen et al., 2013). However, our analysis and other studies in plants (Fait et al., 2020) suggest that stress can also induce a tightening of the network and changes in size. This inconsistency in network patterns could be due to the nature of stressors and the stress sensitivity of the system (Begcy & Dresselhaus, 2018), suggesting plastic adaptive responses to environmental stress.

We identified many highly connected hub genes, related to specific terpene (*CYP*s and *ACT/BAHD*), jasmonate (*LOX*s and *PLD*s), and *MYB* and *WRYK* genes, as it has been documented in several studies of gene expression in defense response pathways (Ling et al., 2020; Pagare et al., 2015). Based on the gene expression profiles of *Datura* species, it is not surprising that these genes showed the highest connectivity and controlled the networks of three of the four species studied. However, the role of specific *TA*s controlling major modules was also revealed for each species. In *D. stramonium* and *D. pruinosa, ODC* and *TRI‐I* tropane genes were highly connected, controlling several *TPS* and *JA*. On the other hand, in *D. wrightii*, *PMT*s and *PAO*s were less connected but coregulate *TPS*. The relationship of genes (nodes) within the networks seems to vary to some extent as a function of the metabolic class. In general, genes implicated in the same biosynthetic pathway were positively correlated, but among pathways, depending on the species, the relationship was more variable, which may suggest differences in ecological and/or physiological constraints to multiple defenses or biosynthetic process (Khare et al., 2020; Sweetlove & Ratcliffe, 2011) and plasticity for restructuring network organization in response to stress (Gaquerel et al., 2014). Overall, our results indicate complex herbivore‐induced networks involving hub genes of key terpene and alkaloid enzymes, signaling by specific jasmonate and transcription factors. All these highly connected genes are good candidates to study the adaptive responses of specialized metabolism and the changes in transcriptional programs in stress response pathways.

## 5 CONCLUSIONS

Secondary metabolism widely varies over the plant lifetime and in response to environmental stress. Metabolic responses are dynamically regulated and include both common and species‐specific mechanisms. Here, we addressed the transcriptional regulation of secondary defensive metabolism in *Datura* species and revealed largely overlapping transcriptional patterns and distinct regulatory responses mediated by developmental and stress‐related programs. Our results indicate differential modulation of terpene and tropane metabolism, which is linked to jasmonate signaling and their regulatory transcription factors. Herbivory induced the transcriptional rearrangement of key *TPS* and *JA*, leading to divergent patterns of coexpression among species. In addition, our data suggest that plant stress responses rely not only on gene/metabolite induction but also on maintaining constitutive levels of defenses. The transcriptional profiles of specialized metabolism shown here will contribute to a better understanding of the molecular basis of adaptive plant responses and the physiological variation of significant ecological traits.

## AUTHOR CONTRIBUTIONS

**Eunice Kariñho Betancourt:** Conceptualization (lead); data curation (lead); formal analysis (lead); investigation (lead); methodology (lead); visualization (lead); writing – original draft (lead). **Nancy Calderón Cortés:** Formal analysis (supporting); software (supporting); validation (supporting); writing – review and editing (supporting). **Rosalinda Tapia López:** Data curation (supporting); methodology (supporting); validation (supporting). **Ivan De‐la‐Cruz:** Formal analysis (supporting); software (supporting). **Juan Núñez Farfán:** Funding acquisition (supporting); investigation (supporting); resources (supporting). **Ken Oyama:** Funding acquisition (lead); investigation (supporting); supervision (lead); writing – review and editing (supporting).

## CONFLICT OF INTEREST STATEMENT

The authors declare no conflict of interest.

## BENEFIT‐SHARING

Benefits from this research accrue from the sharing of our data and results on public databases, as described above.

## Supporting information


Table S1

Table S2

## Data Availability

The datasets presented in this study can be found in online repositories. The names of the repository/repositories and accession number(s) can be found below: https://www.ncbi.nlm.nih.gov/, PRJNA669339.
