# Correction to “Comparative Transcriptome Profiling Reveals Distinct Regulatory Responses of Secondary Defensive Metabolism in Datura Species (Solanaceae) Under Plant Development and Herbivory‐Mediated Stress”

**DOI:** 10.1002/ece3.70409

**Published:** 2024-10-03

Kariñho Betancourt, E., Calderón Cortés, N., Tapia López, R., De‐la‐Cruz, I., Núñez Farfán, J., & Oyama, K. Comparative transcriptome profiling reveals distinct regulatory responses of secondary defensive metabolism in Datura species (Solanaceae) under plant development and herbivory‐mediated stress. Ecology and Evolution 2024; 14(7), e11496.

In the first page, the address of the first author includes ^1^Escuela Nacional de Estudios Superiores (ENES) Unidad Morelia, UNAM, Morelia, Mexico and ^2^Laboratorio de Genética Ecológica y Evolución, Instituto de Ecología, UNAM, Ciudad de México, Mexico, but does not include the current author address, which is essential for the recognition of the institution where the analysis, writing and revision of the final version of the manuscript was carried out over the last year.

The first author and coauthors addresses should have read:

Eunice Kariñho Betancourt^1,2,3^ | Nancy Calderón Cortés^1^ | Rosalinda Tapia López^4^ | Ivan De‐la‐Cruz^5^ | Juan Núñez Farfán^2^ | Ken Oyama^1^

^1^Escuela Nacional de Estudios Superiores (ENES) Unidad Morelia, UNAM, Morelia, Mexico.

^2^Laboratorio de Genética Ecológica y Evolución, Instituto de Ecología, UNAM, Ciudad de México, Mexico.

^3^Current address: Facultad de Estudios Superiores Zaragoza Campus III, UNAM, CP90640, San Miguel Contla, Tlaxcala, Mexico.

^4^Laboratorio de Evolución Molecular y Experimental, Instituto de Ecología, UNAM, Ciudad de México, Mexico.

^5^Department of Plant Protection Biology, Swedish University of Agricultural Sciences, Alnarp, Sweden.

We apologize for this error.

